# Comparison of Radiologic Results after Lateral Meniscal Allograft Transplantation with or without Capsulodesis Using an All-Soft Suture Anchor

**DOI:** 10.3390/medicina59010001

**Published:** 2022-12-20

**Authors:** Dong Ryun Lee, Young Je Woo, Sung Gyu Moon, Woo Jong Kim, Dhong Won Lee

**Affiliations:** 1 Department of Orthopaedic Surgery, KonKuk University Medical Center, Konkuk University School of Medicine, Seoul 05030, Republic of Korea; 2 Department of Radiology, KonKuk University Medical Center, Konkuk University School of Medicine, Seoul 05030, Republic of Korea; 3 Department of Orthopaedic Surgery, Soonchunhyang University Hospital Cheonan, Cheonan 31151, Republic of Korea

**Keywords:** meniscal allograft transplantation, meniscal extrusion, capsulodesis, magnetic resonance imaging

## Abstract

*Background and Objectives*: Studies analyzing magnetic resonance imaging (MRI) after simultaneously performing lateral meniscal allograft transplantation (MAT) and capsulodesis are currently rare. This study aimed to compare the MRI results between the group that performed lateral MAT alone and the group that performed both lateral MAT and capsulodesis simultaneously. *Materials and Methods*: A total of 55 patients who underwent lateral MAT with a 1-year follow-up MRI were included. The patients were divided into two groups according to the surgical procedure: group I (isolated lateral MAT, n = 26) and group C (combined lateral MAT and capsulodesis, n = 29). Differences between groups were compared regarding subjective knee scores, graft extrusion, graft signal, articular cartilage loss, and joint space width (JSW). *Results*: The subjective knee scores improved significantly in both groups (all, *p* < 0.001), and there were no significant differences in these scores between both groups at the 1-year follow-up. Group C showed less coronal graft extrusion at the 1-year follow-up (1.1 ± 1.7 mm vs. 2.4 ± 1.8 mm, *p* < 0.001). Pathologic coronal graft extrusion (≥3 mm) was found in seven (26.9%) patients in group I and three (10.3%) in group C. Concerning the graft signal, group C showed less grade 3 signal intensity in the posterior root of the graft. There were no significant differences in preoperative and postoperative cartilage status between groups. Regarding JSW, there were no significant differences in postoperative JSW between both groups. However, in group C, JSW significantly increased from 3.9 ± 0.4 mm to 4.5 ± 1.4 mm (*p* = 0.031). *Conclusions*: In lateral MAT, capsulodesis (open decortication and suture anchor fixation) could reduce graft extrusion without complications. In the future, large-volume and long-term prospective comparative studies are needed to confirm the clinical effects following capsulodesis.

## 1. Introduction

Meniscal allograft transplantation (MAT) reduces knee pain, improves knee function, and alleviates the degenerative progression of articular cartilage [1,2,3,4,5]. A recent systematic review by Novaretti et al. [6] stated that MAT showed good long-term survivorship rates, with a 10-year survival rate of 73.5% and a 15-year survival rate of 60.3%. However, it has been reported that graft extrusion frequently occurs after MAT, which leads to the abrasion of the articular cartilage and the aggravation of subchondral bone lesions in an inappropriate biomechanical environment [1,5,7,8,9,10].

The causes of graft extrusion after MAT include graft size mismatch before surgery, malposition of the graft during surgery, overtensioning of the meniscal suture, osteophytes, and overstuffing. Graft size reduction, anatomic placement of the graft, and peripheral osteophyte excision have been performed to overcome graft extrusion [11,12,13,14,15,16,17]. Recently, surgeons have been performing capsulodesis, which reduces the space where the graft extrusion may occur by pulling the stretched lateral capsule to the lateral tibial plateau [18,19,20]. The capsulodesis includes a method using a suture anchor and another using transosseous sutures, and each surgeon has reported different results on whether it can reduce graft extrusion [18,19,20].

Studies analyzing magnetic resonance images (MRI) after simultaneously performing lateral MAT and capsulodesis are currently rare. It is believed that different results may be shown depending on the method of performing capsulodesis. Therefore, this study aimed to compare the MRI results between the group that performed lateral MAT alone and the group that performed both lateral MAT and capsulodesis (open decortication and suture anchor fixation) simultaneously. It was hypothesized that the group that simultaneously underwent lateral MAT and capsulodesis would show less graft extrusion and osteoarthritis progression on the MRI one year after MAT.

## 2. Materials and Methods

### 2.1. Patients

**Figure 1:**
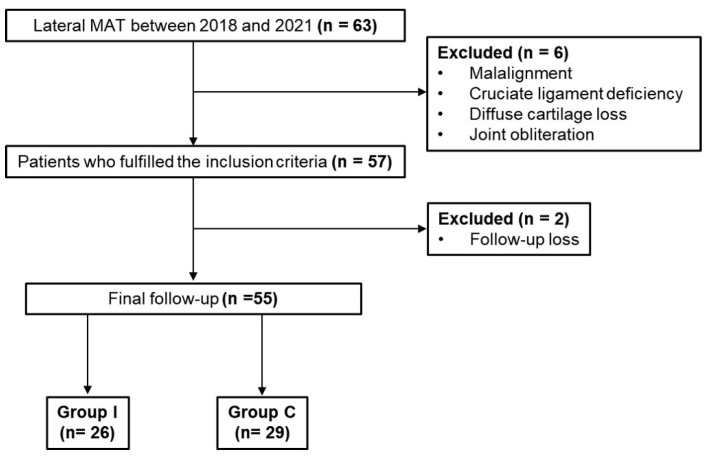
Flowchart of included patients in both groups.

A total of 63 patients who underwent lateral MAT in our institute from March 2018 to July 2021 were retrospectively reviewed. The lateral MAT was indicated for patients with failed conservative treatment after subtotal or total meniscectomy for 6 months and with visual analog scale (VAS) scores ≥ 3 for pain. The patients who underwent MRI examination within 2 days postoperatively and were available for follow-up evaluation for ≥1 year were included. Exclusion criteria were axial limb malalignment ≥ 5°, cruciate ligament deficiency, diffuse osteoarthritis categorized as more than grade 3 according to modified Outerbridge grade (MOG), joint obliteration on the Rosenberg view, and medial compartment osteoarthritis ≥ Kellgren-Lawrence grade 2. However, MAT was performed as a salvage operation in cases of localized MOG 3 or 4 lesions in which coverage by the meniscal graft could be expected. Finally, out of 63 patients, 55 were selected. The patients were divided into two groups according to the surgical procedure in a consecutive and non-random manner: group I (isolated lateral MAT, n = 26) with patients from March 2018 to June 2019, and group C (combined lateral MAT and capsulodesis, n = 29) with patients from July 2019 onward (Figure 1). Combined capsulodesis was started with a clinical suspicion that it could benefit graft stabilization. This retrospective comparative study was conducted following approval from the Ethics Committee of Konkuk University Medical Center (KUMC 2022-09-001).

### 2.2. Surgical Techniques

Lateral MAT was performed using the keyhole technique with modified instruments by a single experienced surgeon (D.W. Lee) [17]. The allografts were fresh-frozen and non-irradiated. After debridement of the peripheral rim of the remaining lateral meniscus, a mini arthrotomy was performed. A keyhole-shaped slot was made using our customized osteotome and dilator along the centers of the anterior and posterior root attachments under the lateral eminence of the tibial articular surface [17]. A posterolateral incision was made for meniscal sutures. To reach the posterolateral area, dissection was carefully performed between the inferior-to-iliotibial band and the superior-to-biceps femoris complex. Through an anteromedial portal, a suture passing wire with the loop positioned posteriorly for the leading suture for traction was passed through the posterolateral capsule in an in-to-outside fashion.

**Figure 2:**
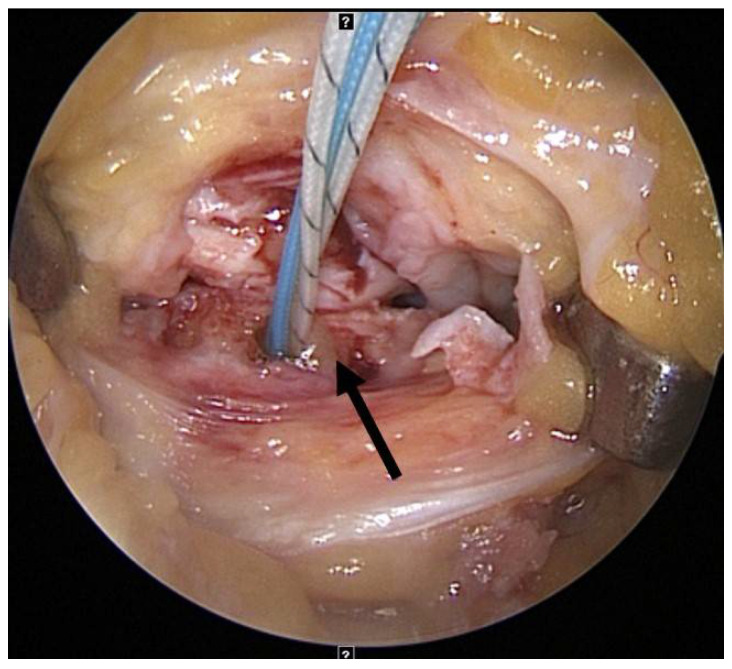
All-soft suture anchor is inserted slightly anterior to the midbody level of the lateral meniscus.

**Figure 3:**
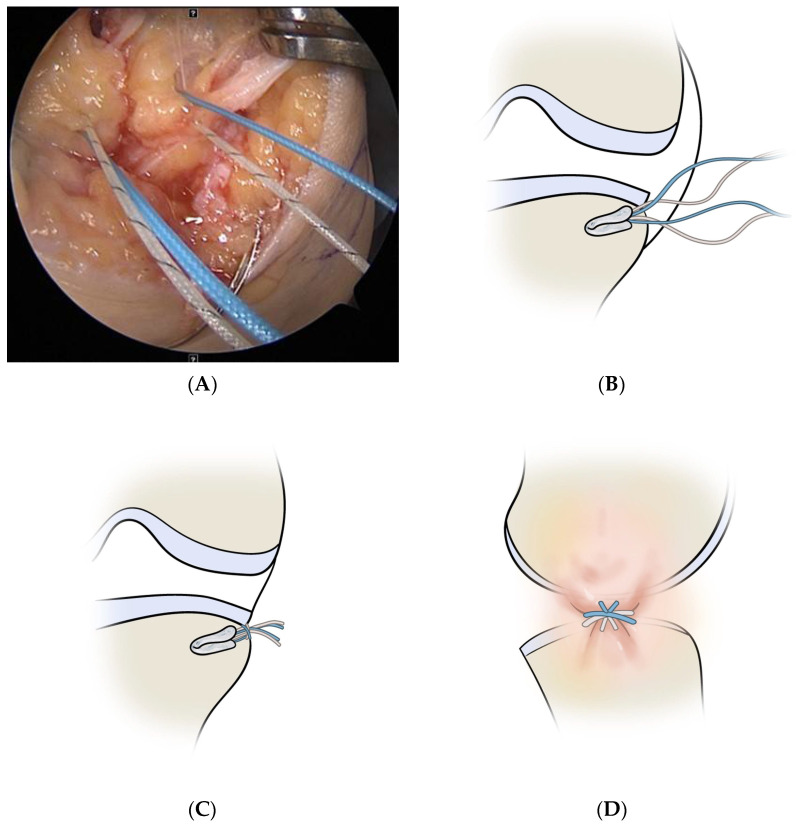
Capsulodesis using an all-soft suture anchor. (**A**,**B**) Two pairs of strands of suture anchor are pulled out of the lateral capsule. (**C**,**D**) They form an X shape and are tied. The lateral capsule is attached to the lateral tibial plateau after capsulodesis.

**Figure 4:**
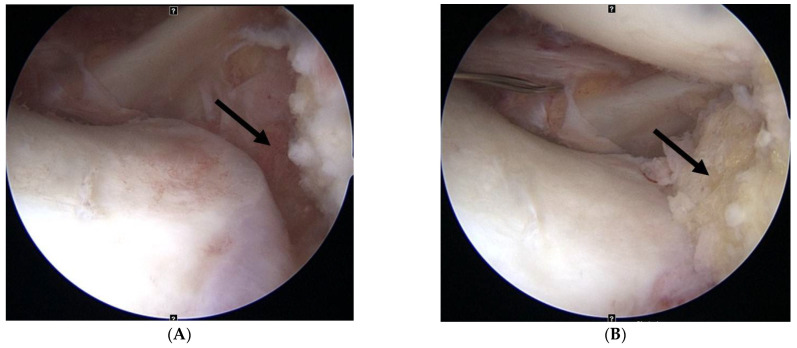
(**A**) The lateral capsule is displaced from the lateral tibial plateau previous to capsulodesis. (**B**) The lateral capsule is attached to the lateral tibial plateau after capsulodesis.

In patients undergoing capsulodesis, after palpating the lateral epicondyle, an oblique small stab wound was made anteriorly on the iliotibial band, and the articular capsule was exposed by bending the iliotibial band up and down. After exposing the rim of the lateral tibial plateau by longitudinally excising the articular capsule, a part of the cartilage and the cortical bone were removed gently in the 10 mm anterior and 10 mm posterior sections using a small rongeur to expose the cancellous bone. At the rim of the lateral tibial plateau, an all-soft suture anchor (Y-Knot, ConMed Linvatec, Largo, FL, USA) was inserted slightly anterior to the midbody level of the lateral meniscus (Figure 2). Two pairs of strands of suture anchor were pulled out of the joint to form an X shape at a point of 5–7 mm at the anterior and posterior margins of the incised articular capsule and then sutured (Figure 3A–D). The narrowing of the lateral space, in which the lateral joint capsule was attached to the lateral tibial plateau, was identified using arthroscopy, and stabilization of the lateral capsule was confirmed with probing (Figure 4). The bone bridge of the allograft was inserted into the keyhole-shaped slot to position the allograft into the joint. After confirming that the anterior and posterior allograft was in the appropriate position, the midbody was sutured using the inside-out method, the posterior horn using the Fast-Fix 360 system (Smith & Nephew Endoscopy, Andover, MA, USA), and the anterior horn using the outside-in method.

For postoperative rehabilitation, the same delayed rehabilitation protocol was applied to all patients [21]. The cast was fixed while applying a varus force for the first three weeks after the surgery, and the range of motion (ROM) exercise was started with the cast-off after three weeks. ROM was targeted at 90 degrees by six weeks and 120 degrees by 12 weeks. A lateral unloading brace (DonJoy OA Adjuster; DJO Global, Vista, CA, USA) was immediately put on while casting off and maintained until 12 weeks after surgery. Crutch walking was started immediately after surgery, and weight bearing was gradually performed until six weeks so that full weight bearing was possible. Isometric quadriceps muscle-strengthening and straight leg-raising exercises were begun immediately after surgery, and isokinetic exercise was performed in weeks 10–12. A gradual functional improvement exercise was performed so that the patients could return to light jogging at 4–5 months, noncontact sports at 7–9 months, and contact sports at 10–12 months. Only patients who met the criteria by performing muscle strength and functional tests were allowed to return to exercise.

### 2.3. Radiological Evaluation

**Figure 5:**
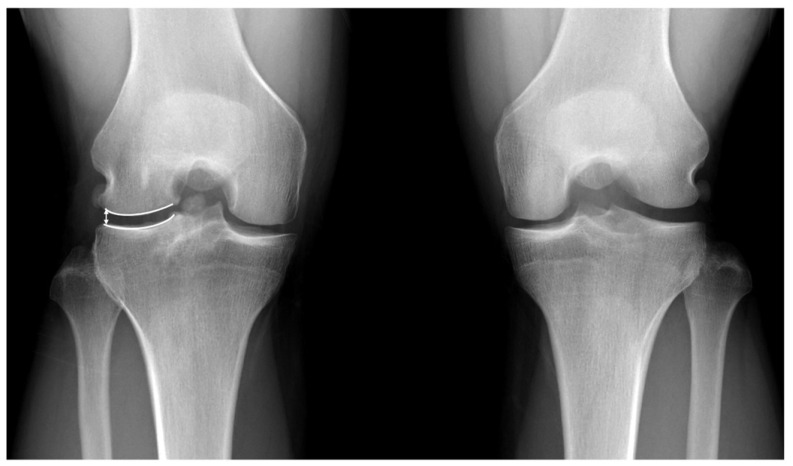
The absolute joint space width is measured from the lateral edge of the femoral condyle to the lateral edge of the tibial plateau (white arrow).

The lateral joint space width (JSW) was measured from the Rosenberg. The measurements were performed on a picture archiving and communication system (PACS) workstation (Centricity RA 1000; GE Healthcare, Chicago, IL, USA). The absolute JSW was calculated from the lateral edge of the femoral condyle to the lateral edge of the tibial plateau [22,23,24] (Figure 5). Our previous study proved that only the lateral edge was observed to be significantly reduced without whole joint obliteration at the time of lateral meniscal allograft transplantation [24]. The relative JSW was calculated by dividing the absolute JSW of the involved knee by the absolute JSW of the uninvolved knee to better standardize the data. The progression of joint space narrowing (JSN) was assessed. Lower extremity alignment was assessed using the mechanical axis of the hip–knee–ankle angle on a standing anteroposterior scanogram.

**Figure 6:**
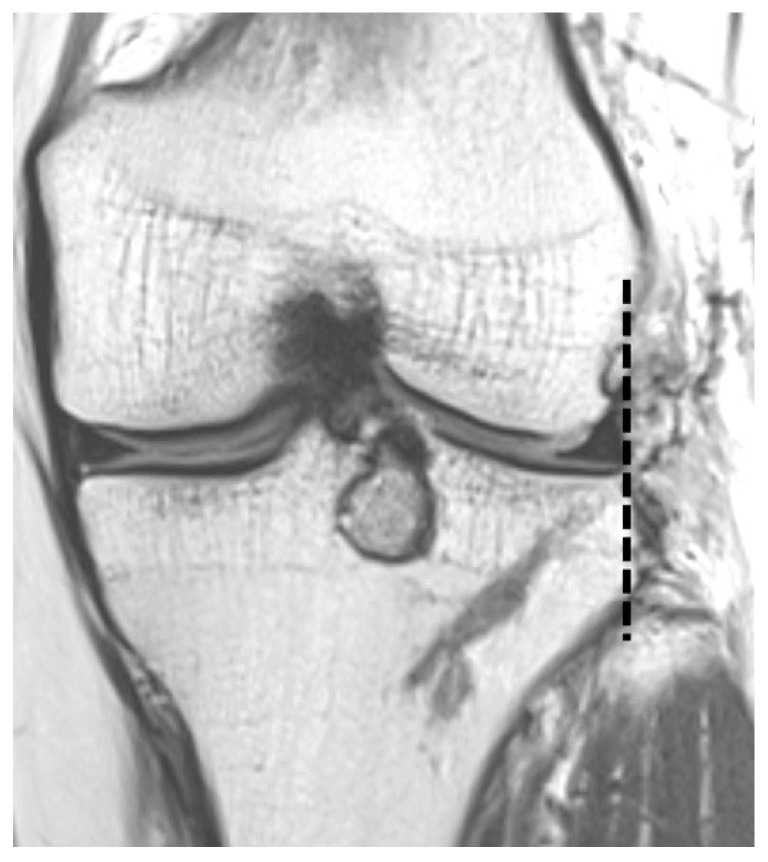
Graft extrusion on the coronal plane is defined as the distance between the lateral margin of the graft and the superolateral aspect of the tibial plateau. There is no graft extrusion.

**Figure 7:**
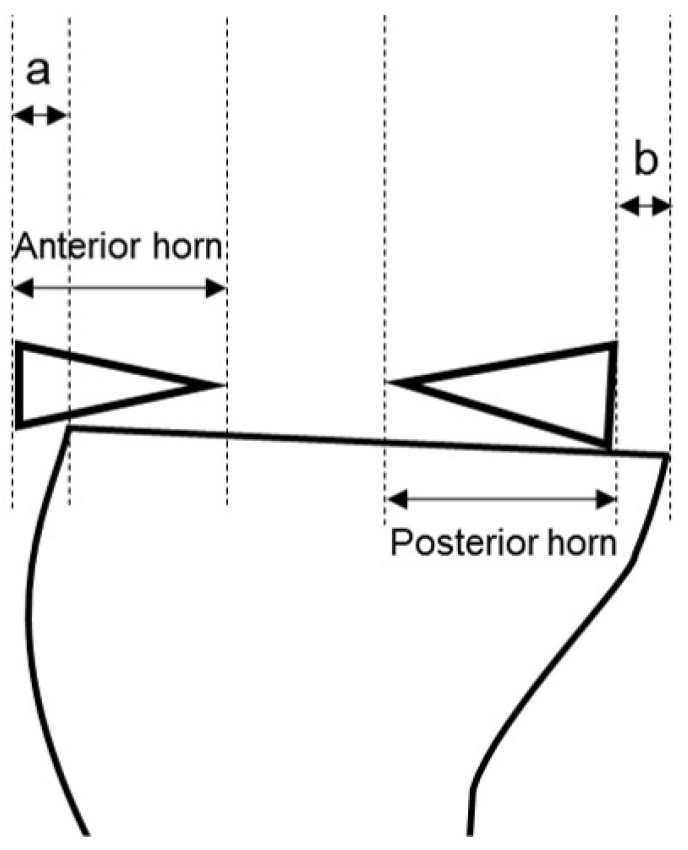
The absolute sagittal anterior horn extrusion (a) and posterior horn extrusion (b).

To evaluate graft position and the status of the cartilage of the lateral compartment, follow-up MRIs at 2 days and 12 months postoperatively were conducted. All patients signed an informed consent form prior to MRI examinations with a 3.0-T system apparatus (Signa HD; GE Healthcare, Milwaukee, WI, USA). Graft extrusion on the coronal plane at the level of the posterior border of the medial collateral ligament was measured from the lateral margin of the graft to the superolateral aspect of the tibial plateau (Figure 6). Pathologic extrusion was defined as graft extrusion ≥ 3 mm [14,25,26,27,28,29]. Graft extrusion on the sagittal plane was measured relative to the anterior horn and posterior horn. The absolute sagittal anterior horn extrusion was defined as the distance from the anterior margin of the proximal tibial articular surface to the anterior border of the anterior horn, and the relative sagittal anterior horn extrusion was calculated as the absolute anterior horn extrusion divided by the entire anterior horn [30] (Figure 7a). The posterior horn extrusion was similarly assessed (Figure 7b). Extrusion of the anterior horn outside the anterior border and of the posterior horn outside the posterior border of the tibial articular cartilage was defined as positive, whereas extrusion inside these borders was defined as negative. The axial trough angle on the axial plane was measured between the line drawn along the long axis of the keyhole-shaped slot and the line perpendicular to the trans posterior tibial condylar line [14,15,27].

**Figure 8:**
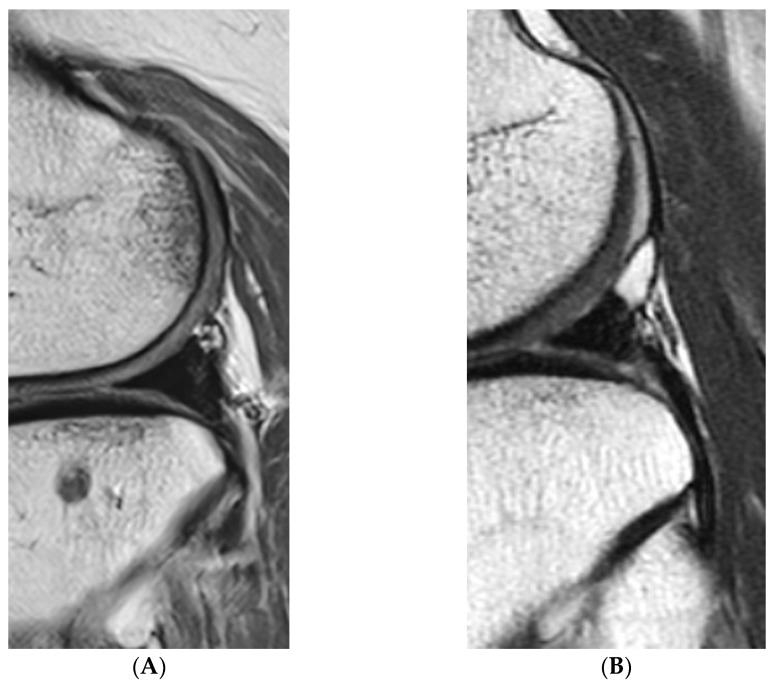

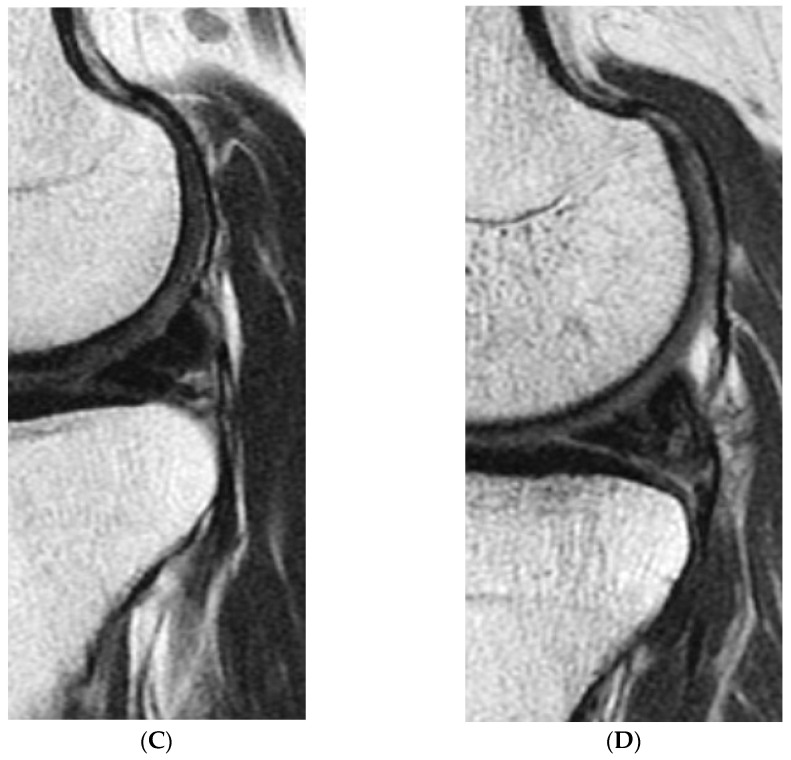
Graft signal. (**A**) Grade 0, (**B**) Grade 1, (**C**) Grade 2, and (**D**) Grade 3.

Graft signal was assessed using MRI at 12 months, postoperatively, and graded following Park et al. [31]: grade 0 (normal), grade 1 (globular increased signal intensity), grade 2 (linear signal intensity within the meniscal graft), and grade 3 (diffuse increased signal intensity or communicated to the articular surface) (Figure 8). This grading was applied for five sections: anterior root, anterior third of the meniscal graft, mid-body, posterior third of the meniscal graft, and posterior root.

The cartilage status at the lateral compartment was assessed according to the MOG (grade 0, normal; grade 1, cartilage surface fibrillation; grade 2, <50% loss of cartilage thickness; grade 3, ≥50% loss of cartilage thickness; grade 4, exposed subchondral bone) [32,33,34]. The worst MOG of the lateral femoral condyle (LFC) and lateral tibial plateau (LTP) was used to present the overall status of the lateral compartment [35]. The cartilage status was categorized into three groups: low-grade (MOG ≤ 2) cartilage lesions on LFC or/and LTP; high-grade (MOG ≥ 3) cartilage lesions on either LFC or LTP; and high-grade cartilage lesions on both LFC and LTP [31].

Radiological measurements were independently performed by one experienced orthopedic surgeon and one experienced radiologist (D.W. Lee and S.G. Moon). Each examiner measured twice with a 6-week interval, and the mean value was used. In the grading system, if there were different results between the two examiners, the results were determined based on consensus.

### 2.4. Clinical Evaluation

The clinical outcomes were evaluated using the Lysholm score and International Knee Documentation Committee (IKDC) subjective knee score. Preoperative data and postoperative data at 12 months were compared.

### 2.5. Statistical Analysis 

Statistical analysis was performed using the SPSS software (IBM SPSS Statistics 21; IBM Corp, Somers, NY, USA). In all analyses, statistical significance was set at *p* < 0.05. The independent t-test or Mann–Whitney U test was used to compare parametric variables between the two groups. Preoperative and postoperative parametric or non-parametric variables were compared using the paired t-test or Wilcoxon signed-rank test in each group. The intraobserver and interobserver reliabilities of measurements were presented with the intraclass correlation coefficient (ICC) [36]. To detect a difference of 2 mm in graft extrusion between group I and group C with a significance level of 5% and a power of 80%, the required sample size was 16 patients for each group. Therefore, the number of patients in the current study had sufficient statistical power [21,24].

## 3. Results

Preoperative demographic data showed no statistically significant differences between both groups and are summarized in Table 1. The Lysholm and IKDC subjective knee scores improved significantly in both groups (from 64.3 ± 10.1 and 55.5 ± 9.8 to 86.4 ± 7.2 and 82.2 ± 8.5 in group I, and from 65.7 ± 8.4 and 54.3 ± 10.7 to 87.3 ± 7.4 and 84.5 ± 8.1 in group C, all *p* < 0.001). There were no significant differences in these scores between both groups at the 1-year follow-up (*p* = 0.650 and 0.309, respectively).

**Figure 9:**
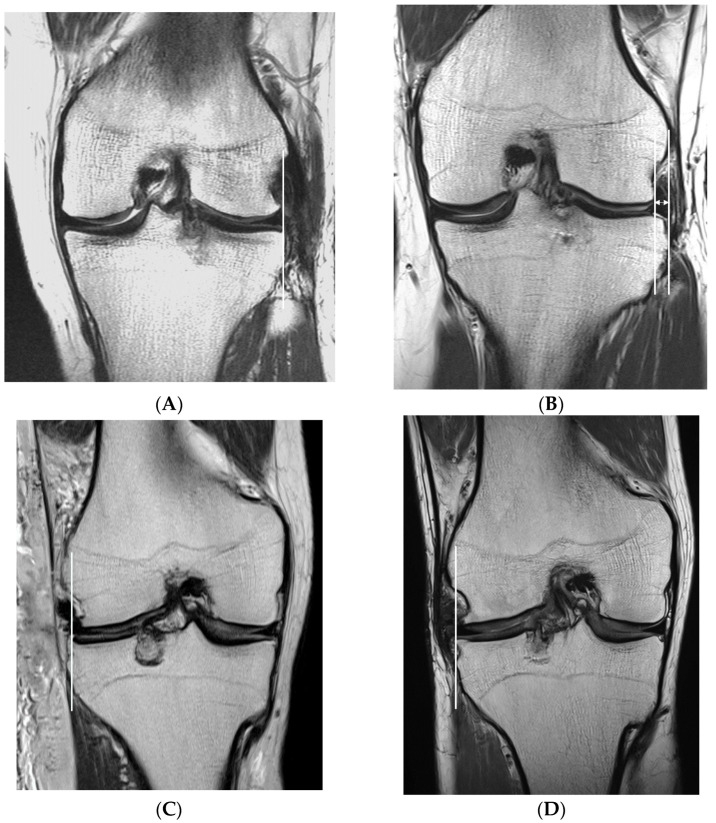
(**A**) Postoperative magnetic resonance image (MRI) at 2 days after lateral meniscal allograft transplantation (MAT) and (**B**) postoperative MRI at 1 year after the surgery in the patient who performed isolated lateral MAT. Graft extrusion increased from 0mm to 2.9mm. (**C**) Postoperative MRI at 2 days after lateral MAT and (**D**) postoperative MRI at 1 year after the surgery in the patient who performed lateral MAT + capsulodesis. Graft extrusion was not found until 1 year after the surgery.

All ICC values for intraobserver and interobserver reliabilities were >0.81 in radiologic measurements, which was considered to be excellent. Regarding the initial graft position verified by MRI 2 days after MAT, there were no significant differences at the axial trough angle, coronal position of the graft, and sagittal position of the anterior and posterior horns of the graft (Table 2). However, group C showed less coronal graft extrusion at the 1-year follow-up (1.1 ± 1.7 mm vs. 2.4 ± 1.8 mm, *p* < 0.001). Coronal graft extrusion increased significantly from 1.4 ± 1.6 mm to 2.7 ± 1.2 in group I (*p* < 0.001) and not in group C (*p* = 0.223) (Figure 9). Pathologic coronal graft extrusion (≥3 mm) was found in seven (26.9%) patients in group I and three (10.3%) in group C. In graft signal on MRI at 1-year follow-up, group C showed less grade 3 signal intensity in the posterior root of the graft (Table 3).

There were no significant differences in preoperative and postoperative cartilage status between both groups (Table 4). Regarding JSW, there were no significant differences in postoperative JSW between both groups (Table 5). However, in group C, JSW significantly increased from 3.9 ± 0.4 mm to 4.5 ± 1.4 mm (*p* = 0.031).

There were no complications related to the suture anchor. Group C showed no lateral capsular tear, meniscocapsular separation, or lateral tibial plateau bone edema.

## 4. Discussion

The main finding of the current study was that combined lateral MAT and capsulodesis (open decortication and suture anchor fixation) showed less coronal graft extrusion and a greater increase of lateral JSW at the 1-year follow-up compared with isolated lateral MAT. Additional capsulodesis also positively affected graft maturation in the posterior root.

Various efforts have been implemented to reduce graft extrusion after MAT, and recently, studies have reported positive results by applying a method of stabilizing the stretched lateral capsule during MAT. Masferrer-Pino et al. [19] found that major graft extrusion greater than 3 mm occurred in 73.3% of the group (n = 15) that performed the suture-only technique and 28.6% of the group (n = 14) that performed capsulodesis (trans-osseous suture). Masferrer-Pino et al. [20] also reported that major graft extrusion greater than 3 mm occurred in 41.4% of the group (n = 15) that performed the bony fixation technique and 53.3% of the group (n = 14) that performed capsulodesis (trans-osseous suture). Seo et al. [18] reported that when comparing the isolated lateral MAT group (n = 13) and the lateral MAT + capsulodesis using the suture anchor group (n = 10), the mean of graft extrusion in the MRI six months after surgery was 1.2 ± 2.1 mm and 2.6 ± 1.3 mm, respectively. The graft extrusion of the isolated MAT group increased by an average of 1.3 mm six months after surgery compared to before surgery, whereas that of the lateral MAT + capsulodesis group decreased by an average of 1.1 mm. In the present study, the coronal graft position was appropriately located in both groups without significant differences in MRI performed on the second day after surgery. The mean graft extrusion in group C was significantly smaller in MRI performed the first year after lateral MAT. In group I, the graft extrusion increased significantly from 0.8 ± 1.6 mm on the second day after surgery to 2.4 ± 1.8 mm in the first year after surgery, and in group C, it was found to be from 0.4 ± 0.9 mm to 1.1 ± 1.7 mm, which was non-significant. Pathologic graft extrusion occurred in 26.9% of the patients in group I and 10.3% in group C. It is thought that lateral capsular healing and graft incorporation could be promoted because the articular capsule was incised to diffusely decorate the rim of the tibial plateau before performing a procedure for attaching the lateral capsule to it, and capsulodesis was performed with a wide X-shaped attachment.

The cause of high signal intensity seen in MRI after MAT is believed to be extracellular matrix degeneration or fibrocartilaginous scar formation rather than tear [31,37]. The clinical significance of high signal intensity has not yet been elucidated. When the signal intensity was evaluated for the five sections of the graft on MRI one year after surgery in this study, there was a significant difference between the two groups in the posterior root section, and the grade 3 high signal intensity showed a high frequency in group I. As the cause of graft immaturity in the posterior root in group I, it can be considered that stress was more concentrated in the posterior root section, which played an essential role in load distribution due to greater graft extrusion compared to group C [28,38]. Park et al. [31] explained that when 138 MRIs were evaluated three years or more after MAT, grade 3 high signal intensity was observed in approximately one-third of them; in particular, the case of distorted contour with a grade 3 high signal intensity in the posterior third and posterior root was significantly associated with inferior outcomes.

We did not apply universal classification such as Kellgren-Lawrence grade to determine joint space narrowing of the lateral edge [39,40]. Lee et al. [24] revealed that joint cartilage is relatively well maintained when only the lateral edge, not the joint obliteration, is narrow in a considerable number of patients. They showed no significant differences in the ratio of lesions ≥ modified Outerbridge grade 3 between less and moderate joint space narrowing groups. Thus, we suggest that joint space narrowing of the lateral edge and cartilage destruction are not closely related. For this reason, there seems to be no difference in subjective clinical scores at short-term follow-up between the two groups in the current study. Moreover, we assume that the degree of graft extrusion in the first year after lateral MAT minimally affected the subjective clinical outcomes [5,21,41].

Capsulodesis may be an excellent attempt to reduce graft extrusion after MAT, but further biomechanical studies are needed to elucidate how this procedure will affect the physiological meniscal movement. Seo et al. [18] stated that one capsular tear and three meniscocapsular separations occurred among ten patients who underwent MAT + capsulodesis. They hypothesized that the occurrence of these complications might be a limitation of normal meniscal excrusion due to capsulodesis. In this study, no capsular tear or meniscocapsular separation occurred, which is thought to be the difference in the type of suture anchor and the suture method.

The current technique for lateral capsulodesis using an all-soft suture anchor seems to be a simple, reliable, and easy method to prevent graft extrusion. There is also less possibility of complications due to additional procedures. The all-soft suture anchor did not result in significant bone loss in the lateral tibial plateau and allowed for optimal postoperative MRI evaluation.

There are several limitations in this study. First, two different surgical methods were not randomly assigned because lateral capsulodesis was started with clinical suspicion that it could be beneficial for graft stabilization. A lateral MAT was not conducted frequently under strict criteria, and the current study had a relatively small sample size and could not perform a prospective randomized trial. However, the inclusion criteria for both groups were the same, and preoperative demography after retrospective analysis revealed that the two groups did not differ significantly. Second, since the result of MRI and clinical examinations in the first year are considered in this study, a longer follow-up is necessary to identify how graft extrusion affects long-term clinical outcomes. Third, it may be necessary to use two suture anchors, depending on the size of the tibia, but this study conducted capsulodesis using only one. Fourth, objective functional tests were not performed. It is necessary to analyze how graft extrusion affects motor function in closed kinetic chain situations as well as subjective function scores. Finally, there was no second-look arthroscopy; thus, the authors could not evaluate intra-articular biological conditions.

## 5. Conclusions

In lateral MAT, capsulodesis (open decortication and suture anchor fixation) could reduce graft extrusion without complications. In the future, large-volume and long-term prospective comparative studies are needed to confirm the clinical effect following capsulodesis.

## Figures and Tables

**Table 1:** Demographic data.

	Group I (n = 26)	Group C (n = 29)	*p*-Value
Age (yrs)	31.9 ± 8.3	32.6 ± 12.1	0.806
Sex (Male/Female)	14/12	16/13	0.772
Body Mass Index, kg/m^2^	23.1 ± 4.2	22.8 ± 3.7	0.779
Period from meniscectomy to MAT, months	18.2 ± 7.3	17.3 ± 6.8	0.638
MRI follow-up duration, months	12.7 ± 1.8	13.2 ± 2.1	0.350
Preoperative Lysholm score	64.3 ± 10.1	65.7 ± 8.4	0.577
Preoperative IKDC subjective score	55.5 ± 9.8	54.3 ± 10.7	0.668
Preoperative mechanical axis (hip-knee-ankle), °	0.3 ± 1.7	0.1 ± 1.9	0.684
Preoperative JSW of lateral edge, mm	3.7 ± 0.5	3.9 ± 0.4	0.106
Preoperative MOG, n (%) Low grade High grade on either LFC or LTP	23 (88.5%) 2 (7.7%)	25 (86.2%) 3 (10.3%)	
High grade on both LFC and LTP	1 (3.8%)	1 (3.4%)	

MAT, meniscal allograft transplantation; JSW, joint space width; MOG, modified Outerbridge grade; MRI, magnetic resonance imaging.

**Table 2:** Graft extrusion.

	Group I (n = 26)	Group C (n = 29)	*p*-Value
Aixal trough angle (°) on MRI postoperative 2 days	4.3 ± 2.7	4.6 ± 2.2	0.652
Sagittal graft anterior horn extrusion on MRI postoperative 2 days			
Absolute value, mm Relative value, %	1.3 ± 1.6 14.5 ± 8.1	1.4 ± 1.8 13.7 ± 8.4	0.829 0.721
Sagittal graft posterior horn extrusion on MRI postoperative 2 days			
Absolute value, mm Relative value, %	−1.3 ± 1.0 9.9 ± 7.8	−1.1 ± 1.2 10.3 ± 7.1	0.508 0.843
Coronal graft extrusion on MRI postoperative 2 days, mm ^a^	0.8 ± 1.6	0.4 ± 0.9	0.252
Coronal graft extrusion on MRI postoperative 1 year, mm ^b^	2.4 ± 1.8	1.1 ± 1.7	<0.001
*p*-value *	<0.001	0.055	

MRI, magnetic resonance imaging. * Paired *t*-test (a and b).

**Table 3:** Graft Signal on MRI at 1-year follow-up.

Signal Intensity	Group I (n = 26)	Group C (n = 29)	*p*-Value
Anterior root, n (%) Grade 0–2 Grade 3 Anterior one-third, n (%) Grade 0–2 Grade 3 Mid-body, n (%) Grade 0–2 Grade 3 Posterior one-third, n (%) Grade 0–2 Grade 3 Posterior root, n (%) Grade 0–2 Grade 3	20 (76.9%) 6 (23.1%) 22 (84.6%) 4 (15.3%) 23 (88.5%) 3 (11.5%) 21 (80.8%) 5 (19.2%) 19 (73.1%) 7 (26.9%)	27 (93.1%) 2 (6.9%) 28 (96.6%) 1 (3.4%) 29 (100%) 0 28 (96.6%) 1 (3.4%) 28 (96.6%) 1 (3.4%)	0.131 0.178 0.099 0.090 0.020

**Table 4:** Cartilage status.

	Group I (n = 26)	Group C (n = 29)	*p*-Value
Preoperative MOG, n (%) Low grade High grade on either LFC or LTP High grade on both LFC and LTP	23 (88.5%) 2 (7.7%) 1 (3.8%)	25 (86.2%) 3 (10.3%) 1 (3.4%)	0.941
At 1-year follow-up MOG, n (%) Low grade High grade on either LFC or LTP High grade on both LFC and LTP	23 (88.5%) 2 (7.7%) 1 (3.8%)	28 (96.6%) 1 (3.4%) 0	0.404

MOG, modified Outerbridge grade; low grade: MOG grade 0, 1, or 2; high grade: MOG 3 or 4; LFC, lateral femoral condyle; LTP, lateral tibial plateau.

**Table 5:** Changes in Joint Space Width of Lateral Edge on Rosenberg View.

	Group I (n =26)	Group C (n =29)	*p*-Value
Contralateral JSW, mm Preoperative 1 year postoperatively	5.6 ± 2.3 5.5 ± 2.4	5.7 ± 2.5 5.7 ± 2.8	0.878 0.779
*p*-value	0.879	0.892	
Absolute JSW, mm Preoperative 1 year postoperatively	3.7 ± 0.5 4.0 ± 1.8	3.9 ± 0.4 4.5 ± 1.4	0.106 0.253
*p*-value	0.417	0.031	
Relative JSW Preoperative 1 year postoperatively	0.7 ± 0.5 0.7 ± 0.7	0.7 ± 0.8 0.8 ± 0.9	0.738 0.602
*p*-value	0.889	0.656	

## Data Availability

The data that support the findings of this study are available on request from the corresponding author.
